# Perceived Morbidity, Healthcare-Seeking Behavior and Their Determinants in a Poor-Resource Setting: Observation from India

**DOI:** 10.1371/journal.pone.0125865

**Published:** 2015-05-12

**Authors:** Suman Kanungo, Kalyan Bhowmik, Tanmay Mahapatra, Sanchita Mahapatra, Uchhal K. Bhadra, Kamalesh Sarkar

**Affiliations:** 1 National Institute of Cholera and Enteric Diseases, Kolkata, 700010, West Bengal, India; 2 Medical College, Malda, 732101, West Bengal, India; Texas Tech University Health Science Centers, UNITED STATES

## Abstract

**Background:**

To control the double burden of communicable and non-communicable diseases (NCDs), in the developing world, understanding the patterns of morbidity and healthcare-seeking is critical. The objective of this cross-sectional study was to determine the distribution, predictors and inter-relationship of perceived morbidity and related healthcare-seeking behavior in a poor-resource setting.

**Methods:**

Between October 2013 and July 2014, 43999 consenting subjects were recruited from 10107 households in Malda district of West Bengal state in India, through multistage random sampling, using probability proportional-to-size. Information on socio-demographics, behaviors, recent ailments, perceived severity and healthcare-seeking were analyzed in SAS-9.3.2.

**Results:**

Recent illnesses were reported by 55.91% (n=24600) participants. Among diagnosed ailments (n=23626), 50.92% (n=12031) were NCDs. Respiratory (17.28%,n=7605)), gastrointestinal (13.48%,n=5929) and musculoskeletal (6.25%,n=2749) problems were predominant. Non-qualified practitioners treated 53.16% (n=13074) episodes. Older children/adolescents [adjusted odds ratio for private healthcare providers(AOR_Pri_)=0.76, 95% confidence interval=0.71-0.83) and for Govt. healthcare provider(AOR_Govt_)=0.80(0.68-0.95)], females [AOR_Govt_=0.80(0.73-0.88)], Muslims [AOR_Pri_=0.85(0.69-0.76) and AOR_Govt=_0.92(0.87-0.96)], backward castes [AOR_Govt_=0.93(0.91-0.96)] and rural residents [AOR_Pri_=0.82(0.75-0.89) and AOR_Govt_=0.72(0.64-0.81)] had lower odds of visiting qualified practitioners. Apparently less severe NCDs [acid-peptic disorders: AOR_Pri_=0.41(0.37-0.46) & AOR_Govt_=0.41(0.37-0.46), osteoarthritis: AOR_Pri_=0.72(0.59-0.68) & AOR_Govt_=0.58(0.43-0.78)], gastrointestinal [AOR_Pri_=0.28(0.24-0.33) & AOR_Govt_=0.69(0.58-0.81)], respiratory [AOR_Pri_=0.35(0.32-0.39) & AOR_Govt_=0.46(0.41-0.52)] and skin infections [AOR_Pri_=0.65(0.55-0.77)] were also less often treated by qualified practitioners. Better education [AOR_Pri_=1.91(1.65-2.22) for ≥graduation], sanitation [AOR_Pri_=1.58(1.42-1.75)] and access to safe water [AOR_Pri_=1.33(1.05-1.67)] were associated with healthcare-seeking from qualified private practitioners. Longstanding NCDs [chronic obstructive pulmonary diseases: AOR_Pri_=1.80(1.46-2.23), hypertension: AOR_Pri_=1.94(1.60-2.36), diabetes: AOR_Pri_=4.94(3.55-6.87)] and serious infections [typhoid: AOR_Pri_=2.86(2.04-4.03)] were also more commonly treated by qualified private practitioners. Potential limitations included temporal ambiguity, reverse causation, generalizability issues and misclassification.

**Conclusion:**

In this poor-resource setting with high morbidity, ailments and their perceived severity were important predictors for healthcare-seeking. Interventions to improve awareness and healthcare-seeking among under-privileged and vulnerable population with efforts to improve the knowledge and practice of non-qualified practitioners probably required urgently.

## Introduction

Demographic ageing, unplanned urbanization and unhealthy lifestyles are the major contributors for the changing pattern of disease in recent years, from communicable to non-communicable diseases (NCDs), globally.[1–3] This epidemiological transition is spreading fast in the developing world, progressively affecting poor, vulnerable and disadvantaged populations.[3,4] Nearly 80% of the current burden of NCDs like cardio-vascular disease, diabetes, cancer and chronic respiratory diseases occurred in low and middle-income countries (LMIC), accounting for 90% of premature (< 60 years) deaths.[1,4,5] As major fraction of this global burden of disease was attributed to preventable risk factors, known behavioral and medical interventions could prevent about 80% of these premature deaths.[3,6] In this era of changing epidemiological trend, the scenario is worsening gradually in LMICs including India where increasing mortality and morbidity are attributable to double burden of communicable and non-communicable diseases in poor-resource settings.[7–9]

Despite remarkable progress in socio-economic development and having an overarching aim of addressing the health needs through several comprehensive programs, health outcomes in India remained poor. During 2012, approximately 60% deaths were attributed to NCDs (cardiovascular diseases = 26%, chronic respiratory diseases = 13%, cancers = 7%, diabetes = 2% & injuries = 12%) and 28% to communicable, maternal, perinatal and nutritional conditions in this country.[10,11] Evidences suggested that healthcare infrastructure, service delivery system and health outcomes varied considerably across Indian states and for efficient improvement of these parameters, understanding the morbidity patterns and their predictors seemed to be required urgently.[12] It has also been established in recent past that self-perceived morbidity is a reliable measure for estimating the burden especially in a poor-resource setting.[13–16]

Individual healthcare-seeking pattern in a community is determined by complex interrelationships between socio-economic and physical environment along with individual characteristics and behaviors.[17] Thus healthcare-seeking pattern and related outcomes have been the focus of community level improvement of health systems worldwide and India is no exception. In last few years, studies have shown that household information based on door-to-door visits were useful for the identification of gaps in perceived morbidity and resultant healthcare-seeking in both urban and rural areas.[18,19] Diverse healthcare-seeking patterns, especially involving non-qualified practitioners and pharmacists often resulted in inadequate treatment, improper dosing and over-the-counter purchase of drugs, frequently culminating into development of antimicrobial resistance and other unfavorable outcomes.[20–22]

Relevant researches on morbidity and healthcare-seeking ever conducted in India were mostly limited to urban areas of southern and western part while eastern region remained largely understudied.[23] Malda is one of the poorest districts, situated in the north-eastern part of the state of West Bengal, India; sharing interstate borders with Bihar and Jharkhand, and international border with Bangladesh. Thus international and interstate migration resulted in uneven demographic pressures on the healthcare infrastructure that had to cater 1,870 populations per hospital bed.[24] The district health situation urgently demanded appropriately targeted public health interventions for mitigation of gaps and up-gradation of the healthcare infrastructure to achieve proper control of communicable and non-communicable diseases. For this purpose, proper understanding of the perceived morbidity, related healthcare-seeking and their predictors among residents of this district seemed to be the need of the hour.

Hence, a community-based cross-sectional study was designed involving a representative population of Malda to understand the distribution of the perceived morbidity and healthcare-seeking behavior, their predictors and inter-relationship.

## Methods

### Ethics Statement

The study protocol was reviewed and approved by the Ethics Committee of the National Institute of Cholera and Enteric Diseases, Kolkata. Written informed consent left thumb impression (for illiterates, in presence of two impartial literate witnesses) was obtained from residents older than 18 years and from the guardians of residents aged 1 to 17 years. Written assent was additionally obtained from residents aged 12 to 17 years.

### Recruitment

Based on the 2011 census data, the urban area of the Malda district was divided into two broad urban administrative divisions termed as Municipalities (Old Malda and English Bazar). Each Municipality was further subdivided into smaller administrative units called Wards (19 in Old Malda and 25 in English Bazar). Using probability proportional to size (PPS) determined by the total number of households in the Wards, 4 Wards in Old Malda and 12 Wards in English Bazar were selected randomly. The rural area of the district consisted of 3701 villages and 27 rural towns from which similarly using PPS, 25 villages/census towns were selected randomly. Using an exhaustive house-list of the urban and rural areas, each selected municipal ward and village/rural town was categorized into several segments (considered as Primary Sampling Unit: PSU), each having 125 households (defined as those who shared the cooking-pot in each dwelling). Next, 4012 urban and 6095 rural households (maintaining the population ratio) were selected from the whole district, through multistage random sampling, using PPS. Thus, 16 municipal wards in urban and 24 villages/towns in rural area were selected. In each selected ward/village from the list of segments two were selected randomly and all households were surveyed there after collecting written informed consent from the residents.

### Interview

All the individuals residing in the selected households were interviewed at home by trained interviewers, using a structured, pre-tested, bi-lingual (English and local language: Bengali) questionnaire. Information was collected on socio-demographic and related variables such as age, gender, religion, caste, education level and occupation of the household members, maximum education level among adults in the house, house ownership, residential area, type and location of water source, water treatment at home, material used for cooking and domestic light source. Housing type was classified as Kachha (if neither roof/walls/floors was made of permanent materials like bricks/cement/stone), Pacca (if roof, walls and floors all were made of permanent materials like bricks/cement/stone) and Semi-pacca (for any combinations between Kaccha and Pacca builts regarding roof, walls and floors). Sanitation level of toilet use practices were categorized as poor (if the household had no toilet and the members used open space/field/jungle for defecation), good (for households having toilets with flush to piped sewer system/flush to septic tank) and all others (flush to pit latrine/flush to elsewhere/all other types of pit latrine etc.) as average.

Based on the information regarding household assets (enquired using an appropriate list of assets), number of cattle, goats/sheep, poultry, place for keeping them and the aforementioned household information, wealth index was calculated by using relative weights for each and then the cumulative wealth index scores were log-transformed and divided into quintiles of socio-economic status: SES (very poor, poor, lower middle, upper middle and upper) based on the percentile distribution.

For all the members of the selected households, information regarding last three episodes of ailments that forced them to seek some healthcare services within last two months was collected. Occurrence, perceived severity and healthcare-seeking behavior (visited non-qualified/qualified private sector/qualified Govt. sector practitioners) regarding specific NCDs like: acid peptic disorder (APD) or peptic ulcer disorder (PUD), chronic obstructive pulmonary disease (COPD), hypertension (HTN), diabetes mellitus (DM), anemia and osteoarthritis (OA) as well as communicable diseases like: gastroenteritis, respiratory tract infection (RTI), typhoid and skin infections were also collected.

### Data analyses

Thus between October 2013 and July 2014, 43999 individuals (with approximately 8% non-response) were recruited from 10107 households (4012 urban and 6095 rural) and collected data were analyzed using Statistical Analysis System (SAS) version 9.3.2. Distribution of the socio-demographic characteristics, morbidity pattern and healthcare-seeking were determined by conducting descriptive analyses using survey frequency procedure to determine overall and stratified frequencies, proportions and corresponding 95% confidence intervals (95%CI). Bivariate and multivariate logistic regression analyses were next conducted to determine unadjusted (OR) and adjusted (for age, gender, religion, caste, individual and familial education, occupational type, residential area, sanitation and SES) odds ratios (AOR) as the measures of association (with corresponding 95%CIs) between study variables. Multinomial logistic regressions [25] were used where the dependent variables had more than two categories.

## Results

Among 43999 subjects, majorities were aged 18–40 yrs (40.74%, n = 17925), male (50.65%, n = 22287), Hindu (67.89%, n = 29869), general caste (42.11%, n = 18526) and educated up to secondary level (33.44%, n = 12782). For 38.82% (n = 17080). Maximum adult education in the household was also up to secondary level, 95.73% (n = 42122) stayed in own house, 39.60% (n = 15888) were in sedentary work and 62.60% (n = 27543) lived in rural areas. (Table 1)

**Table 1:** Overall and stratified (across the strata of health-seeking behavior) distribution of socio-demographic characteristics among recruited residents of Malda, West Bengal, India (N = 43999).

Socio-demographics	Categories	Total (N = 43999)		Didn’t report any recent morbidity (n = 19404)		Reported to have recent morbidity & care sought from (Practitioner type)					
						Non-qualified (13074)		Qualified, private sector (8368)		Qualified, Govt. sector (3153)	
		n	Percentage (95%CI)	n	Percentage (95%CI)	n	Percentage (95%CI)	n	Percentage (95%CI)	n	Percentage (95%CI)
Age group of the subject	<5 years	3873	8.80(8.54–9.07)	1298	6.69(6.34–7.04)	1375	10.52(9.99–11.04)	967	11.56(10.87–12.24)	233	7.39(6.48–8.30)
	5–<18 years	12043	27.37(26.95–27.79)	7008	36.12(35.44–36.79)	3013	23.05(22.32–23.77)	1359	16.24(15.45–17.03)	663	21.03(19.6–22.45)
	18–40 years	17925	40.74(40.28–41.20)	8484	43.72(43.02–44.42)	5208	39.83(39.00–40.67)	3073	36.72(35.69–37.76)	1160	36.79(35.11–38.47)
	41–60 years	7911	17.98(17.62–18.34)	2154	11.10(10.66–11.54)	2741	20.97(20.27–21.66)	2195	26.23(25.29–27.17)	821	26.04(24.51–27.57)
	>60 years	2247	5.11(4.90–5.31)	460	2.37(2.16–2.58)	737	5.64(5.24–6.03)	774	9.25(8.63–9.87)	276	8.75(7.77–9.74)
Gender	Male	22287	50.65(50.19–51.12)	10624	54.75(54.05–55.45)	6073	46.45(45.60–47.31)	4000	47.8(46.73–48.87)	1590	50.43(48.68–52.17)
	Female	21712	49.35(48.88–49.81)	8780	45.25(44.55–45.95)	7001	53.55(52.69–54.40)	4368	52.2(51.13–53.27)	1563	49.57(47.83–51.32)
Religion	Hindu	29869	67.89(67.45–68.32)	12843	66.19(65.52–66.85)	8860	67.77(66.97–68.57)	6011	71.83(70.87–72.80)	2155	68.35(66.72–69.97)
	Muslim	13975	31.76(31.33–32.20)	6498	33.49(32.82–34.15)	4158	31.8(31.01–32.60)	2335	27.90(26.94–28.87)	984	31.21(29.59–32.83)
	Christian	144	0.33(0.27–0.38)	58	0.30(0.22–0.38)	53	0.41(0.30–0.51)	22	0.26(0.15–0.37)	11	0.35(0.14–0.55)
	Sikh	11	0.03(0.01–0.04)	5	0.03(0.00–0.05)	3	0.02(0.00–0.05)	-	-	3	0.10(0.00–0.20)
Caste	Scheduled caste	16104	36.60(36.15–37.05)	6918	35.65(34.98–36.33)	4962	37.95(37.12–38.79)	2889	34.52(33.51–35.54)	1335	42.34(40.62–44.07)
	Scheduled tribe	1589	3.61(3.44–3.79)	709	3.65(3.39–3.92)	623	4.77(4.40–5.13)	182	2.18(1.86–2.49)	75	2.38(1.85–2.91)
	Other backward class	7780	17.68(17.33–18.04)	3611	18.61(18.06–19.16)	2141	16.38(15.74–17.01)	1499	17.91(17.09–18.74)	529	16.78(15.47–18.08)
	General	18526	42.11(41.64–42.57)	8166	42.08(41.39–42.78)	5348	40.91(40.06–41.75)	3798	45.39(44.32–46.45)	1214	38.50(36.80–40.20)
Education level of the subject	Illiterate	9557	25.00(24.57–25.44)	3075	17.87(17.3–18.44)	3693	33.29(32.42–34.17)	1795	25.24(24.23–26.25)	994	35.39(33.62–37.16)
	Primary	11916	31.17(30.71–31.64)	5856	34.03(33.32–34.73)	3462	31.21(30.35–32.07)	1756	24.69(23.69–25.69)	842	29.98(28.28–31.67)
	Secondary	12782	33.44(32.97–33.91)	6210	36.08(35.37–36.8)	3223	29.06(28.21–29.90)	2564	36.05(34.94–37.17)	785	27.95(26.29–29.61)
	Higher-secondary	2086	5.46(5.23–5.69)	1069	6.21(5.85–6.57)	404	3.64(3.29–3.99)	486	6.83(6.25–7.42)	127	4.52(3.75–5.29)
	Graduation and above	1882	4.92(4.71–5.14)	1000	5.81(5.46–6.16)	310	2.79(2.49–3.10)	511	7.19(6.58–7.79)	61	2.17(1.63–2.71)
Maximum educational level among adult members in the household	Illiterate	6838	15.54(15.20–15.88)	2740	14.12(13.63–14.61)	2611	19.97(19.29–20.66)	881	10.53(9.87–11.19)	606	19.22(17.84–20.60)
	Primary	9130	20.75(20.37–21.13)	3939	20.3(19.73–20.87)	3130	23.94(23.21–24.67)	1345	16.07(15.29–16.86)	716	22.71(21.25–24.17)
	Secondary	17080	38.82(38.36–39.27)	7556	38.94(38.25–39.63)	5020	38.40(37.56–39.23)	3291	39.33(38.28–40.38)	1213	38.47(36.77–40.17)
	Higher-secondary	4957	11.27(10.97–11.56)	2315	11.93(11.47–12.39)	1188	9.09(8.59–9.58)	1121	13.40(12.67–14.13)	333	10.56(9.49–11.63)
	Graduation and above	5994	13.62(13.30–13.94)	2854	14.71(14.21–15.21)	1125	8.60(8.12–9.09)	1730	20.67(19.81–21.54)	285	9.04(8.04–10.04)
House ownership	Owned	42122	95.73(95.55–95.92)	18661	96.17(95.9–96.44)	12533	95.86(95.52–96.20)	7951	95.02(94.55–95.48)	2977	94.42(93.62–95.22)
	Rented	1421	3.23(3.06–3.39)	558	2.88(2.64–3.11)	399	3.05(2.76–3.35)	338	4.04(3.62–4.46)	126	4.00(3.31–4.68)
	Others	456	1.04(0.94–1.13)	185	0.95(0.82–1.09)	142	1.09(0.91–1.26)	79	0.94(0.74–1.15)	50	1.59(1.15–2.02)
Occupational type	Sedentary	15888	39.60(39.12–40.07)	8531	47.12(46.39–47.84)	3828	32.72(31.87–33.57)	2534	34.24(33.16–35.32)	995	34.08(32.36–35.80)
	Moderate worker	12907	32.17(31.71–32.62)	4746	26.21(25.57–26.85)	4097	35.02(34.16–35.88)	3032	40.97(39.85–42.09)	1032	35.34(33.61–37.08)
	Hard Worker	11331	28.24(27.80–28.68)	4829	26.67(26.03–27.31)	3774	32.26(31.41–33.11)	1835	24.79(23.81–25.78)	893	30.58(28.91–32.25)
Residential area	Rural	27543	62.60(62.15–63.05)	12192	62.83(62.15–63.51)	8959	68.53(67.73–69.32)	4475	53.48(52.41–54.55)	1917	60.80(59.09–62.50)
	Urban	16456	37.40(36.95–37.85)	7212	37.17(36.49–37.85)	4115	31.47(30.68–32.27)	3893	46.52(45.45–47.59)	1236	39.20(37.5–40.91)
Water source	Unsafe	1455	3.31(3.14–3.47)	678	3.49(3.24–3.75)	426	3.26(2.95–3.56)	238	2.84(2.49–3.20)	113	3.58(2.93–4.23)
	May be unsafe	40208	91.38(91.12–91.65)	17671	91.07(90.67–91.47)	12258	93.76(93.34–94.17)	7375	88.13(87.44–88.83)	2904	92.10(91.16–93.04)
	Safe	2336	5.31(5.10–5.52)	1055	5.44(5.12–5.76)	390	2.98(2.69–3.27)	755	9.02(8.41–9.64)	136	4.31(3.60–5.020)
Location of water source	Elsewhere	22140	50.32(49.85–50.79)	9657	49.77(49.06–50.47)	6497	49.69(48.84–50.55)	4394	52.51(51.44–53.58)	1592	50.49(48.75–52.24)
	In own yard/plot	15209	34.57(34.12–35.01)	6757	34.82(34.15–35.49)	4649	35.56(34.74–36.38)	2684	32.07(31.07–33.07)	1119	35.49(33.82–37.16)
	In own dwelling	6650	15.11(14.78–15.45)	2990	15.41(14.90–15.92)	1928	14.75(14.14–15.35)	1290	15.42(14.64–16.19)	442	14.02(12.81–15.23)
Water treatment at home	No	41825	95.06(94.86–95.26)	18391	94.78(94.47–95.09)	12689	97.06(96.77–97.35)	7719	92.24(91.67–92.82)	3026	95.97(95.29–96.66)
	Yes	2174	4.94(4.74–5.14)	1013	5.22(4.91–5.53)	385	2.94(2.66–3.23)	649	7.76(7.18–8.33)	127	4.03(3.34–4.71)
Sanitation level of the practices regarding toilet use	Poor	11856	26.95(26.53–27.36)	5133	26.45(25.83–27.07)	4282	32.75(31.95–33.56)	1523	18.20(17.37–19.03)	918	29.12(27.53–30.70)
	Average	18668	42.43(41.97–42.89)	8287	42.71(42.01–43.40)	5634	43.09(42.24–43.94)	3338	39.89(38.84–40.94)	1409	44.69(42.95–46.42)
	Good	13475	30.63(30.20–31.06)	5984	30.84(30.19–31.49)	3158	24.15(23.42–24.89)	3507	41.91(40.85–42.97)	826	26.20(24.66–27.73)
Material used for cooking	Crop residue/Cow dung cake	13441	30.55(30.12–30.99)	6084	31.36(30.71–32.01)	4610	35.27(34.45–36.09)	1900	22.71(21.81–23.61)	847	26.86(25.32–28.41)
	Firewood/Coal/lignite/charcoal	17376	39.50(39.04–39.96)	7624	39.30(38.61–39.99)	5525	42.27(41.42–43.12)	2758	32.97(31.96–33.97)	1469	46.59(44.85–48.33)
	Kerosene	379	0.86(0.78–0.95)	156	0.80(0.68–0.93)	120	0.92(0.75–1.08)	73	0.87(0.67–1.07)	30	0.95(0.61–1.29)
	LPG/PNG/Electricity	12794	29.08(28.66–29.51)	5536	28.54(27.90–29.17)	2816	21.54(20.84–22.25)	3635	43.45(42.39–44.51)	807	25.59(24.07–27.12)
Housing type	Kachha	15377	34.97(34.52–35.41)	6808	35.10(34.43–35.78)	5260	40.26(39.42–41.10)	2114	25.28(24.34–26.21)	1195	37.90(36.21–39.59)
	Semi-pucca	16639	37.84(37.38–38.29)	7152	36.88(36.20–37.56)	5133	39.29(38.45–40.12)	3023	36.14(35.11–37.17)	1331	42.21(40.49–43.94)
	Pacca	11961	27.20(26.78–27.61)	5434	28.02(27.39–28.65)	2673	20.46(19.77–21.15)	3227	38.58(37.54–39.63)	627	19.89(18.49–21.28)
Light source at the household	No lighting	62	0.14(0.11–0.18)	26	0.13(0.08–0.19)	20	0.15(0.09–0.22)	8	0.10(0.03–0.16)	8	0.25(0.08–0.43)
	Kerosene	4802	10.92(10.62–11.21)	2032	10.47(10.04–10.90)	1754	13.42(12.83–14.00)	585	6.99(6.44–7.54)	431	13.67(12.47–14.87)
	Solar	32	0.07(0.05–0.10)	14	0.07(0.03–0.11)	9	0.07(0.02–0.11)	7	0.08(0.02–0.15)	2	0.06(0.00–0.15)
	Electricity	39098	88.87(88.58–89.17)	17330	89.32(88.89–89.76)	11288	86.36(85.77–86.95)	7768	92.83(92.28–93.38)	2712	86.01(84.80–87.22)
Socio-economic status (SES)	Very poor	9186	20.88(20.50–021.26)	3657	18.85(18.30–19.40)	3452	26.40(25.65–27.16)	1288	15.39(14.62–16.17)	789	25.02(23.51–26.54)
	Poor	10157	23.08(22.69–23.48)	4216	21.73(21.15–22.31)	3085	23.60(22.87–24.32)	2022	24.16(23.25–25.08)	834	26.45(24.91–27.99)
	Lower middle	7065	16.06(15.71–16.40)	3112	16.04(15.52–16.55)	1948	14.90(14.29–15.51)	1513	18.08(17.26–18.91)	492	15.60(14.34–16.87)
	Upper middle	9038	20.54(20.16–20.92)	4182	21.55(20.97–22.13)	2338	17.88(17.23–18.54)	1991	23.79(22.88–24.71)	527	16.71(15.41–18.02)
	Upper	8553	19.44(19.07–19.81)	4237	21.84(21.25–22.42)	2251	17.22(16.57–17.86)	1554	18.57(17.74–19.40)	511	16.21(14.92–17.49)

n = Stratum specific number of participants; 95%CI = 95% Confidence Interval

Only 5.31% (n = 2336) were drinking safe water, 50.32% (n = 22140) had to bring drinking water from outside, 95.06% (n = 41825) were not doing any water treatment at home, 29.08% (n = 12794) were using gas/electricity for cooking, 27.20% (n = 11961) were living in pacca houses. Electricity was the source of lighting at home for 88.87% (n = 39098), regarding toilet use 30.63% (n = 13475) had good sanitary practices and overall 19.44% (n = 8553) belonged to upper SES. Overall and stratified (across healthcare-seeking patterns) socio-demographic distribution are presented in Table 1.

Regarding the distribution of self-perceived most recent (within past 2 month) morbidity, 44.09% (n = 19399) did not suffer from any such recently while for 17.28% (n = 7605), 13.48% (n = 5929) and 6.25% (n = 2749) residents the most recent morbidity was related to respiratory, gastrointestinal and musculoskeletal system respectively. Among the most recent ailments, NCDs were 50.92% (n = 12031), 53.16% (n = 13074) episodes were treated by non-qualified practitioners, 34.02% (n = 8368) by qualified practitioner from private sector and only 12.82% (n = 3153) by qualified practitioner from Govt. sector. Non-qualified practitioners were treating more communicable diseases compared to NCDs [57.52% (n = 7194) vs. 42.48% (n = 5313)]. (Table 2)

**Table 2:** Overall and stratified (across the strata of health-seeking behavior) distribution of self-perceived morbidities among recruited residents of Malda, West Bengal, India (N = 43999).

Distribution of all types of self-perceived morbidity* (based on most recent ailments)**		Total		Care sought from (Practitioner type)					
				Non-qualified		Qualified, private sector		Qualified, Govt. sector	
		n	Percentage (95%CI)	n	Percentage (95%CI)	n	Percentage (95%CI)	n	Percentage (95%CI)
Organ/System/Function involved	None	19399	44.09(43.63–44.55)						
	Respiratory	7605	17.28(16.93–17.64)	4760	36.41(35.58–37.23)	2031	24.27(23.35–25.19)	814	25.82(24.29–27.35)
	Gastrointestinal	5929	13.48(13.16–13.79)	3416	26.13(25.38–26.88)	1763	21.07(20.19–21.94)	749	23.76(22.27–25.24)
	Musculoskeletal	2749	6.25(6.02–6.47)	1451	11.10(10.56–11.64)	966	11.54(10.86–12.23)	332	10.53(9.46–11.6)
	Hematological/Immunological/Metabolic/Parasitic disorders	1985	4.51(4.32–4.71)	1102	8.43(7.95–8.91)	587	7.01(6.47–7.56)	295	9.36(8.34–10.37)
	Darmatological	1419	3.23(3.06–3.39)	731	5.59(5.20–5.99)	463	5.53(5.04–6.02)	223	7.07(6.18–7.97)
	Hypertension	761	1.73(1.61–1.85)	168	1.29(1.09–1.48)	493	5.89(5.39–6.40)	100	3.17(2.56–3.78)
	Neurological	605	1.38(1.27–1.48)	253	1.94(1.70–2.17)	241	2.88(2.52–3.24)	111	3.52(2.88–4.16)
	Eye/Nose/Throat related	553	1.26(1.15–1.36)	239	1.83(1.60–2.06)	231	2.76(2.41–3.11)	83	2.63(2.07–3.19)
	Reproductive	552	1.25(1.15–1.36)	221	1.69(1.47–1.91)	265	3.17(2.79–3.54)	66	2.09(1.59–2.59)
	Dental	490	1.11(1.02–1.21)	320	2.45(2.18–2.71)	116	1.39(1.14–1.64)	54	1.71(1.26–2.17)
	Ophthalmological	476	1.08(0.99–1.18)	83	0.63(0.50–0.77)	293	3.50(3.11–3.90)	100	3.17(2.56–3.78)
	Diabetes mellitus	374	0.85(0.76–0.94)	38	0.29(0.20–0.38)	282	3.37(2.98–3.76)	54	1.71(1.26–2.17)
	Urological	267	0.61(0.53–0.68)	37	0.28(0.19–0.37)	189	2.26(1.94–2.58)	41	1.30(0.90–1.70)
	Cardiovascular	194	0.44(0.38–0.50)	24	0.18(0.11–0.26)	128	1.53(1.27–1.79)	42	1.33(0.93–1.73)
	Thyroid disorders	178	0.40(0.35–0.46)	8	0.06(0.02–0.10)	152	1.82(1.53–2.10)	17	0.54(0.28–0.79)
	Cancer	67	0.15(0.12–0.19)	31	0.24(0.15–0.32)	26	0.31(0.19–0.43)	10	0.32(0.12–0.51)
	Injury/Bites	53	0.12(0.09–0.15)	12	0.09(0.04–0.14)	13	0.16(0.07–0.24)	28	0.89(0.56–1.22)
	Psychiatric	50	0.11(0.08–0.15)	4	0.03(0.00–0.06)	40	0.48(0.33–0.63)	6	0.19(0.04–0.34)
	Poisoning	2	<0.01(0.00–0.01)	-	-	-	-	2	0.06(0.00–0.15)
Type of morbidity	Communicable diseases	11595	49.08(48.44–49.71)	7194	57.52(56.65–58.39)	2947	36.59(35.53–37.64)	1452	47.47(45.70–49.24)
	Non-communicable diseases	12031	50.92(50.29–51.56)	5313	42.48(41.61–43.35)	5108	63.41(62.36–64.47)	1607	52.53(50.76–54.30)
Treated by	Non-qualified practitioner	13074	53.16(52.53–53.78)						
	Qualified practitioner from private sector	8368	34.02(33.43–34.62)						
	Qualified practitioner from Govt. sector	3153	12.82(12.40–13.24)						
Specific ailments (Based on last three episodes of ill-health)		No. & Percentage of subjects who recently suffered		Care sought from (Practitioner type)					
				Non-qualified		Qualified, private sector		Qualified, Govt. sector	
		n	Percentage (95%CI)	n	Percentage (95%CI)	n	Percentage (95%CI)	n	Percentage (95%CI)
Respiratory tract infection		6734	19.01(18.60–19.42)	4614	50.12(49.1–51.15)	1552	34.67(33.27–36.06)	568	34.76(32.45–37.07)
Peptic ulcer disease/Acid peptic disorder		2554	8.18(7.87–8.48)	1700	18.47(17.68–19.26)	661	14.76(13.72–15.80)	193	11.81(10.25–13.38)
Gastroenteritis		1977	6.45(6.17–6.72)	1337	14.52(13.80–15.24)	367	8.20(7.39–9.00)	273	16.71(14.90–18.52)
Skin infections & related disorder		1070	3.60(3.38–3.81)	595	6.46(5.96–6.97)	315	7.04(6.29–7.79)	160	9.79(8.35–11.23)
Hypertension		793	2.69(2.51–2.88)	184	2.00(1.71–2.28)	510	11.39(10.46–12.32)	99	6.06(4.90–7.22)
Chronic obstructive pulmonary		601	2.05(1.89–2.21)	170	1.85(1.57–2.12)	307	6.86(6.12–7.60)	124	7.59(6.30–8.87)
Osteoarthritis		559	1.91(1.75–2.07)	311	3.38(3.01–3.75)	198	4.42(3.82–5.03)	50	3.06(2.22–3.90)
Diabetes mellitus		408	1.40(1.27–1.54)	44	0.48(0.34–0.62)	311	6.95(6.20–7.69)	53	3.24(2.38–4.10)
Anaemia		365	1.26(1.13–1.38)	192	2.09(1.79–2.38)	128	2.86(2.37–3.35)	45	2.75(1.96–3.55)
Typhoid		255	0.88(0.77–0.99)	58	0.63(0.47–0.79)	128	2.86(2.37–3.35)	69	4.22(3.25–5.20)
Variables	Categories	No. & Percentage of subjects who recently suffered		Treatment supervised by (Practitioner type)					
				Non-qualified		Qualified, private sector		Qualified, Govt. sector	
		n	Percentage (95%CI)	n	Percentage (95%CI)	n	Percentage (95%CI)	n	Percentage (95%CI)
Perceived severity	Easily recovered/ Well controlled	9589	62.61(61.84–63.37)	6493	70.54(69.61–71.47)	2146	47.93(46.47–49.40)	950	58.14(55.75–60.53)
	Partially recovered/not fully controlled	1860	12.14(11.63–12.66)	1161	12.61(11.93–13.29)	502	11.21(10.29–12.14)	197	12.06(10.48–13.64)
	Not Recovered with initial treatment	3867	25.25(24.56–25.94)	1551	16.85(16.08–17.61)	1829	40.85(39.41–42.29)	487	29.80(27.58–32.02)

n = Stratum specific number of participants; 95%CI = 95% Confidence Interval

* Excluding 291 undiagnosed and 683 “others”

** Group totals may not be identical due to missing values

Based on last three healthcare-seeking episodes, among specific ailments (suffered or not), 19.01% (n = 6734) suffered from RTI, 8.18% (n = 2554) had PUD/APD, 6.45% (n = 1977) experienced gastroenteritis while 3.60% (n = 1070) had some skin problems. Among subjects visiting nonqualified practitioners, only 16.85% (n = 1551) perceived their ailments as severe while this fraction for private sector qualified practitioners, was 40.85% (n = 1829). (Table 2)

Association of socio-demographics with morbidity and healthcare-seeking are presented in Tables 3 and 4. Compared to 18–40 years old, subjects aged 5–18 years were less likely to suffer from APD [AOR = 0.24(0.19–0.30)], COPD [AOR = 0.55(0.38–0.81)], HTN [AOR = 0.02(<0.01–0.11)], DM [AOR = 0.02(<0.01–0.15)], anemia [AOR = 0.16(0.09–0.29)] and OA [AOR = 0.13(0.06–0.29)] but more prone to RTI [AOR = 1.13(1.01–1.27)]. Persons aged 41–60 and >60 years had more APD [AOR_41–60_ = 2.01(1.82–2.23), AOR_>60_ = 2.86(2.41–3.39)], COPD [AOR_41–60_ = 4.80 (3.79–6.09), AOR_>60_ = 13.13(9.89–17.44)], HTN [AOR_41–60_ = 12.86(10.29–16.07), AOR_>60_ = 26.28(20.12–34.31)], DM [AOR_41–60_ = 6.82(5.29–8.80), AOR_>60_ = 12.40(8.86–17.35)], OA [AOR_41–60_ = 12.88(9.93–16.71), AOR_>60_ = 18.58(13.36–25.86)], gastroenteritis [AOR_41–60_ = 1.50(1.29–1.75), AOR_>60_ = 2.44(1.92–3.11)] and RTI [AOR_41–60_ = 1.49(1.36–1.62), AOR_>60_ = 1.82(1.56–2.13)].

**Table 3:** Association (both unadjusted and adjusted) of socio-demographic characteristics with self-perceived specific non-communicable morbidities and their severity among recruited residents of Malda, West Bengal, India (N = 43999).

Socio-demographics	Categories	Measurement (Unadj = Bivariate Adj = Multivariate)	Suffering from specific non-communicable ailments (Based on last three episodes of ill-health)												Perceived severity of disease (Ref = Mild)			
			Acid peptic disorder		COPD		Hypertension		Diabetes Mellitus		Anemia		Osteoarthritis		Moderate		Severe	
			OR (95%CI)	p value	OR (95%CI)	p value	OR (95%CI)	p value	OR (95%CI)	p value	OR (95%CI)	p value	OR (95%CI)	p value	OR (95%CI)	p value	OR (95%CI)	p value
Age group of the subject (Ref = <18–40years)	<5 years	Unadj	**0.11(0.07–0.17)**	**<.0001**	**-**	**-**	**0.07(0.01–0.47)**	**0.0069**	**-**	**-**	**0.09(0.03–0.27)**	**<.0001**	**-**	**-**	**0.27(0.22–0.34)**	**<.0001**	**0.65(0.57–0.74)**	**<.0001**
		Adj	-		-	-	-	-	-	-	-	-	-	-	-	-	-	-
	5–<18 years	Unadj	**0.17(0.15–0.21)**	**<.0001**	0.87(0.65–1.17)	0.3613	**0.03(0.01–0.11)**	**<.0001**	**0.03(0.01–0.12)**	**<.0001**	**0.12(0.08–0.19)**	**<.0001**	**0.15(0.07–0.31)**	**<.0001**	**0.39(0.33–0.45)**	**<.0001**	**0.53(0.47–0.60)**	**<.0001**
		Adj	**0.24(0.19–0.30)**	**<.0001**	**0.55(0.38–0.81)**	**0.0021**	**0.02(<0.01–0.11)**	**<.0001**	**0.02(<0.01–0.15)**	**0.0001**	**0.16(0.09–0.29)**	**<.0001**	**0.13(0.06–0.29)**	**<.0001**	**0.33(0.25–0.42)**	**<.0001**	**0.52(0.43–0.62)**	**<.0001**
	41–60 years	Unadj	**1.97(1.80–2.17)**	**<.0001**	**5.11(4.08–6.40)**	**<.0001**	**11.57(9.37–14.28)**	**<.0001**	**6.59(5.18–8.37)**	**<.0001**	0.83(0.64–1.08)	0.1598	**13.01(10.16–16.65)**	**<.0001**	**1.80(1.59–2.03)**	**<.0001**	**2.54(2.30–2.81)**	**<.0001**
		Adj	**2.01(1.82–2.23)**	**<.0001**	**4.80(3.79–6.09)**	**<.0001**	**12.86(10.29–16.07)**	**<.0001**	**6.82(5.29–8.80)**	**<.0001**	0.95(0.72–1.27)	0.7498	**12.88(9.93–16.71)**	**<.0001**	**1.70(1.49–1.95)**	**<.0001**	**2.34(2.10–2.61)**	**<.0001**
	>60 years	Unadj	**2.48(2.13–2.89)**	**<.0001**	**17.12(13.41–21.85)**	**<.0001**	**28.74(22.76–36.29)**	**<.0001**	**11.77(8.77–15.81)**	**<.0001**	**0.46(0.24–0.90)**	**0.0228**	**21.29(15.96–28.41)**	**<.0001**	**3.01(2.51–3.60)**	**<.0001**	**5.02(4.35–5.79)**	**<.0001**
		Adj	**2.86(2.41–3.39)**	**<.0001**	**13.13(9.89–17.44)**	**<.0001**	**26.28(20.12–34.31)**	**<.0001**	**12.40(8.86–17.35)**	**<.0001**	0.53(0.26–1.08)	0.0824	**18.58(13.36–25.86)**	**<.0001**	**2.63(2.14–3.22)**	**<.0001**	**4.25(3.61–5.00)**	**<.0001**
Gender (Ref = Male)	Female	Unadj	**1.66(1.52–1.80)**	**<.0001**	**0.71(0.60–0.84)**	**<.0001**	**1.45(1.26–1.67)**	**<.0001**	**0.76(0.62–0.92)**	**0.0056**	**13.11(8.91–19.29)**	**<.0001**	**2.49(2.08–2.99)**	**<.0001**	**1.18(1.06–1.30)**	**0.0014**	**1.11(1.03–1.19)**	**0.0090**
		Adj	**1.60(1.45–1.77)**	**<.0001**	**0.59(0.48–0.73)**	**<.0001**	**1.53(1.28–1.83)**	**<.0001**	**0.73(0.57–0.92)**	**0.0085**	**16.26(10.75–24.59)**	**<.0001**	**2.58(2.07–3.22)**	**<.0001**	1.10(0.97–1.25)	0.1302	1.07(0.96–1.18)	0.2139
Religion (Ref = Hindu)	Muslim	Unadj	**0.74(0.68–0.82)**	**<.0001**	**0.81(0.67–0.97)**	**0.0191**	**0.46(0.38–0.56)**	**<.0001**	**0.70(0.56–0.87)**	**0.0016**	0.97(0.78–1.21)	0.7997	**0.81(0.67–0.97)**	**0.0254**	**1.32(1.19–1.47)**	**<.0001**	**0.76(0.70–0.83)**	**<.0001**
		Adj	**0.77(0.69–0.87)**	**<.0001**	0.98(0.77–1.25)	0.8697	1.01(0.81–1.27)	0.9039	**1.40(1.06–1.85)**	**0.0174**	0.94(0.71–1.25)	0.6795	0.90(0.71–1.13)	0.3524	**1.37(1.19–1.57)**	**<.0001**	0.98(0.87–1.10)	0.6786
	Others	Unadj	0.53(0.21–1.29)	0.1607	1.38(0.44–4.35)	0.5872	0.31(0.04–2.19)	0.2375	-	-	2.40(0.75–7.61)	0.1385	0.98(0.24–4.00)	0.9805	1.46(0.73–2.93)	0.2874	0.47(0.22–1.00)	0.0513
		Adj	0.67(0.27–1.67)	0.3870	2.63(0.80–8.61)	0.1114	1.15(0.15–8.63)	0.8928	-	-	2.11(0.64–6.99)	0.2227	1.22(0.28–5.32)	0.7901	1.38(0.61–3.14)	0.4396	0.58(0.23–1.44)	0.2396
Caste (Ref = General)	SC/ST/OBC	Unadj	**0.79(0.73–0.86)**	**<.0001**	1.04(0.88–1.22)	0.6611	**0.66(0.57–0.76)**	**<.0001**	**0.71(0.58–0.86)**	**0.0005**	0.83(0.67–1.02)	0.0743	1.03(0.87–1.22)	0.7427	**0.90(0.81–1.00)**	**0.0393**	**0.89(0.82–0.96)**	**0.0017**
		Adj	**0.74(0.67–0.81)**	**<.0001**	1.00(0.82–1.22)	0.9857	**0.82(0.69–0.97)**	**0.0184**	0.95(0.76–1.18)	0.6212	**0.77(0.60–0.98)**	**0.0348**	0.98(0.80–1.20)	0.8228	1.06(0.93–1.21)	0.3630	0.96(0.87–1.06)	0.4100
Education level of the subject (Ref = Illiterate)	Primary	Unadj	**0.46(0.41–0.51)**	**<.0001**	**0.36(0.29–0.44)**	**<.0001**	**0.37(0.30–0.46)**	**<.0001**	**0.53(0.40–0.72)**	**<.0001**	**0.50(0.39–0.66)**	**<.0001**	**0.27(0.21–0.33)**	**<.0001**	**0.50(0.43–0.57)**	**<.0001**	**0.63(0.57–0.70)**	**<.0001**
		Adj	1.02(0.90–1.17)	0.7187	0.84(0.65–1.09)	0.1902	1.12(0.88–1.43)	0.3650	1.25(0.90–1.74)	0.1811	1.07(0.78–1.48)	0.6716	1.10(0.86–1.41)	0.4628	0.85(0.73–1.01)	0.0569	0.96(0.84–1.09)	0.4963
	Secondary	Unadj	**0.55(0.50–0.61)**	**<.0001**	**0.35(0.29–0.44)**	**<.0001**	**0.70(0.59–0.84)**	**<.0001**	0.92(0.71–1.18)	0.4919	**0.53(0.41–0.69)**	**<.0001**	**0.24(0.19–0.30)**	**<.0001**	**0.55(0.48–0.63)**	**<.0001**	**0.82(0.74–0.91)**	**0.0002**
		Adj	1.08(0.94–1.23)	0.2987	0.80(0.61–1.05)	0.1124	**1.47(1.16–1.86)**	**0.0014**	**1.42(1.03–1.95)**	**0.0334**	1.06(0.75–1.51)	0.7279	0.93(0.71–1.23)	0.6287	**0.84(0.71–0.99)**	**0.0416**	0.98(0.86–1.13)	0.8158
	Higher-secondary	Unadj	**0.48(0.39–0.58)**	**<.0001**	**0.28(0.17–0.45)**	**<.0001**	0.85(0.63–1.14)	0.2677	1.16(0.78–1.73)	0.4734	**0.37(0.21–0.66)**	**0.0007**	**0.19(0.11–0.32)**	**<.0001**	**0.49(0.37–0.66)**	**<.0001**	0.88(0.73–1.08)	0.2180
		Adj	0.98(0.76–1.25)	0.8451	**0.55(0.31–0.95)**	**0.0328**	1.35(0.93–1.97)	0.1181	1.33(0.81–2.18)	0.2592	0.99(0.49–2.02)	0.9820	0.68(0.38–1.22)	0.1959	**0.53(0.38–0.75)**	**0.0003**	**0.76(0.59–0.98)**	**0.0308**
	Graduation and above	Unadj	**0.53(0.43–0.65)**	**<.0001**	**0.43(0.29–0.65)**	**<.0001**	**1.31(1.01–1.70)**	**0.0447**	**1.63(1.13–2.36)**	**0.0098**	**0.26(0.13–0.53)**	**0.0002**	**0.14(0.07–0.26)**	**<.0001**	**0.55(0.41–0.74)**	**0.0001**	**1.32(1.09–1.60)**	**0.0054**
		Adj	1.04(0.79–1.37)	0.7804	0.81(0.47–1.41)	0.4625	**1.63(1.11–2.39)**	**0.0119**	1.43(0.86–2.37)	0.1716	0.98(0.40–2.45)	0.9730	0.53(0.26–1.08)	0.0800	**0.58(0.39–0.85)**	**0.0053**	0.85(0.65–1.11)	0.2313
Maximum educational level among adult household members (Ref = Illiterate)	Primary	Unadj	0.88(0.77–1.01)	0.0680	**0.76(0.58–0.99)**	**0.0442**	0.96(0.68–1.36)	0.8082	1.43(0.86–2.39)	0.1718	0.99(0.72–1.38)	0.9733	**0.67(0.50–0.88)**	**0.0050**	0.89(0.76–1.04)	0.1430	**1.19(1.05–1.36)**	**0.0088**
		Adj	**0.83(0.71–0.98)**	**0.0241**	0.78(0.57–1.05)	0.1025	0.88(0.60–1.29)	0.5239	1.22(0.70–2.11)	0.4863	0.94(0.65–1.37)	0.7515	**0.69(0.50–0.95)**	**0.0217**	0.99(0.82–1.20)	0.9135	**1.25(1.06–1.47)**	**0.0072**
	Secondary	Unadj	0.93(0.82–1.05)	0.2368	**0.79(0.63–1.00)**	**0.0459**	**1.75(1.31–2.34)**	**0.0002**	**2.45(1.56–3.85)**	**<.0001**	0.87(0.65–1.17)	0.3661	**0.76(0.60–0.97)**	**0.0280**	**0.87(0.76–1.00)**	**0.0492**	**1.32(1.18–1.49)**	**<.0001**
		Adj	**0.68(0.58–0.80)**	**<.0001**	**0.69(0.52–0.92)**	**0.0103**	0.91(0.65–1.28)	0.5823	1.18(0.71–1.96)	0.5207	0.81(0.56–1.19)	0.2878	**0.70(0.52–0.93)**	**0.0131**	0.86(0.72–1.02)	0.0889	1.15(0.98–1.34)	0.0853
	Higher-secondary	Unadj	0.91(0.78–1.07)	0.2391	0.80(0.59–1.08)	0.1492	**2.75(2.00–3.78)**	**<.0001**	**3.64(2.24–5.92)**	**<.0001**	0.70(0.47–1.06)	0.0932	0.89(0.65–1.21)	0.4473	1.17(0.97–1.41)	0.0975	**1.86(1.60–2.16)**	**<.0001**
		Adj	**0.57(0.47–0.70)**	**<.0001**	**0.60(0.41–0.88)**	**0.0084**	0.83(0.56–1.23)	0.3516	1.14(0.65–2.01)	0.6514	0.69(0.41–1.16)	0.1596	**0.61(0.42–0.88)**	**0.0080**	1.06(0.84–1.35)	0.6239	**1.41(1.16–1.72)**	**0.0007**
	Graduation and above	Unadj	1.00(0.86–1.16)	0.9991	0.80(0.60–1.06)	0.1225	**5.18(3.88–6.93)**	**<.0001**	**6.36(4.04–10.00)**	**<.0001**	**0.45(0.29–0.71)**	**0.0005**	0.96(0.72–1.28)	0.7988	1.14(0.95–1.37)	0.1683	**2.78(2.42–3.19)**	**<.0001**
		Adj	**0.57(0.46–0.70)**	**<.0001**	**0.54(0.36–0.81)**	**0.0030**	1.08(0.73–1.58)	0.7081	1.47(0.84–2.58)	0.1791	**0.48(0.26–0.87)**	**0.0162**	**0.64(0.44–0.94)**	**0.0227**	0.95(0.74–1.22)	0.6801	**1.54(1.26–1.88)**	**<.0001**
Occupational type (Ref = Sedentary)	Moderate worker	Unadj	**4.24(3.78–4.74)**	**<.0001**	1.13(0.93–1.38)	0.2099	**1.97(1.69–2.31)**	**<.0001**	**2.67(2.11–3.39)**	**<.0001**	**6.45(4.67–8.91)**	**<.0001**	**3.14(2.53–3.89)**	**<.0001**	**1.90(1.67–2.16)**	**<.0001**	**1.64(1.49–1.80)**	**<.0001**
		Adj	**1.61(1.39–1.87)**	**<.0001**	0.78(0.60–1.00)	0.0525	**0.81(0.66–0.99)**	**0.0349**	1.27(0.95–1.69)	0.1063	1.26(0.81–1.96)	0.2978	0.91(0.70–1.19)	0.5019	0.87(0.72–1.05)	0.1416	0.90(0.78–1.04)	0.1514
	Hard Worker	Unadj	**2.75(2.44–3.11)**	**<.0001**	1.00(0.82–1.23)	0.9855	**0.72(0.59–0.89)**	**0.0026**	**1.43(1.08–1.88)**	**0.0112**	**3.43(2.42–4.87)**	**<.0001**	**1.96(1.54–2.48)**	**<.0001**	**1.84(1.61–2.10)**	**<.0001**	**1.14(1.03–1.27)**	**0.0105**
		Adj	**1.45(1.24–1.71)**	**<.0001**	**0.53(0.40–0.69)**	**<.0001**	**0.60(0.46–0.77)**	**0.0001**	0.84(0.60–1.17)	0.3041	**1.89(1.17–3.04)**	**0.0089**	0.90(0.66–1.21)	0.4701	**0.78(0.64–0.95)**	**0.0151**	**0.78(0.67–0.91)**	**0.0017**
Residential area (Ref = Urban)	Rural	Unadj	**0.68(0.63–0.74)**	**<.0001**	**0.77(0.66–0.91)**	**0.0020**	**0.25(0.21–0.29)**	**<.0001**	**0.37(0.31–0.46)**	**<.0001**	0.85(0.69–1.05)	0.1382	1.10(0.92–1.31)	0.2958	**1.35(1.21–1.50)**	**<.0001**	**0.62(0.57–0.67)**	**<.0001**
		Adj	0.95(0.85–1.07)	0.3945	0.86(0.67–1.10)	0.2187	**0.54(0.43–0.67)**	**<.0001**	0.92(0.69–1.22)	0.5461	0.88(0.66–1.18)	0.3886	**1.47(1.15–1.87)**	**0.0019**	**1.39(1.19–1.63)**	**<.0001**	**0.87(0.77–0.98)**	**0.0231**
Water source (Ref = Unsafe)	May be unsafe	Unadj	**1.98(1.47–2.67)**	**<.0001**	1.05(0.67–1.65)	0.8233	**4.21(1.88–9.42)**	**0.0005**	**4.44(1.42–13.85)**	**0.0103**	1.31(0.70–2.47)	0.4003	1.54(0.88–2.67)	0.1288	**1.74(1.21–2.51)**	**0.0028**	0.89(0.72–1.11)	0.2944
		Adj	**1.59(1.17–2.16)**	**0.0032**	0.83(0.52–1.33)	0.4374	2.10(0.92–4.81)	0.0780	2.89(0.91–9.18)	0.0714	1.28(0.67–2.45)	0.4587	1.58(0.89–2.80)	0.1213	**1.75(1.19–2.58)**	**0.0045**	0.83(0.64–1.07)	0.1451
	Safe	Unadj	**2.19(1.56–3.07)**	**<.0001**	1.21(0.70–2.09)	0.5014	**14.69(6.46–33.39)**	**<.0001**	**13.27(4.15–42.39)**	**<.0001**	1.04(0.47–2.31)	0.9152	1.51(0.78–2.90)	0.2208	1.05(0.66–1.69)	0.8252	**2.11(1.64–2.73)**	**<.0001**
		Adj	1.39(0.97–1.99)	0.0743	0.84(0.46–1.54)	0.5705	**2.59(1.10–6.12)**	**0.0297**	**3.52(1.06–11.64)**	**0.0396**	1.27(0.55–2.95)	0.5745	1.50(0.74–3.04)	0.2620	1.08(0.65–1.80)	0.7595	1.16(0.84–1.58)	0.3729
Sanitation level regarding toilet use (Ref = Poor)	Average	Unadj	**1.18(1.06–1.32)**	**0.0025**	1.10(0.89–1.35)	0.3709	**2.37(1.79–3.14)**	**<.0001**	**2.46(1.64–3.67)**	**<.0001**	0.79(0.62–1.01)	0.0585	1.10(0.89–1.36)	0.3819	1.07(0.95–1.21)	0.2722	**1.13(1.02–1.24)**	**0.0158**
		Adj	1.12(0.99–1.26)	0.0766	1.11(0.87–1.42)	0.4098	**1.40(1.02–1.91)**	**0.0375**	**1.74(1.13–2.66)**	**0.0116**	0.84(0.63–1.12)	0.2294	1.16(0.91–1.48)	0.2219	1.07(0.93–1.24)	0.3302	0.92(0.81–1.04)	0.1827
	Good	Unadj	**1.57(1.41–1.76)**	**<.0001**	1.20(0.97–1.49)	0.0919	**6.95(5.33–9.06)**	**<.0001**	**7.53(5.15–11.00)**	**<.0001**	**0.74(0.57–0.97)**	**0.0296**	1.23(0.98–1.53)	0.0752	**1.23(1.08–1.41)**	**0.0018**	**2.14(1.94–2.36)**	**<.0001**
		Adj	**1.34(1.15–1.55)**	**0.0001**	1.10(0.81–1.49)	0.5611	**2.07(1.47–2.92)**	**<.0001**	**3.73(2.37–5.86)**	**<.0001**	0.74(0.52–1.06)	0.1024	**1.38(1.02–1.85)**	**0.0362**	**1.52(1.27–1.83)**	**<.0001**	**1.38(1.19–1.61)**	**<.0001**
Socio-economic status (Ref = Very poor)	Poor	Unadj	**1.21(1.07–1.37)**	**0.0027**	1.03(0.82–1.31)	0.7839	**2.12(1.64–2.74)**	**<.0001**	**1.85(1.28–2.66)**	**0.0010**	0.95(0.72–1.25)	0.7015	1.04(0.80–1.36)	0.7708	1.14(0.97–1.33)	0.1067	**1.16(1.04–1.30)**	**0.0079**
		Adj	1.06(0.93–1.22)	0.3688	1.00(0.76–1.31)	0.9817	1.11(0.84–1.48)	0.4590	0.93(0.63–1.37)	0.7206	1.07(0.79–1.44)	0.6692	1.00(0.75–1.33)	0.9980	1.19(1.00–1.42)	0.0523	0.96(0.83–1.10)	0.5194
	Lower middle	Unadj	1.14(1.00–1.31)	0.0572	0.88(0.67–1.15)	0.3441	**2.40(1.84–3.14)**	**<.0001**	**2.43(1.68–3.52)**	**<.0001**	0.81(0.59–1.12)	0.1993	0.96(0.72–1.30)	0.8016	**1.19(1.00–1.42)**	**0.0461**	**1.46(1.29–1.65)**	**<.0001**
		Adj	1.04(0.90–1.20)	0.6175	0.86(0.63–1.17)	0.3356	1.08(0.80–1.47)	0.6145	1.03(0.69–1.53)	0.8912	0.97(0.69–1.37)	0.8702	0.89(0.65–1.23)	0.4732	**1.25(1.03–1.52)**	**0.0265**	1.16(1.00–1.35)	0.0515
	Upper middle	Unadj	1.02(0.90–1.16)	0.7501	0.92(0.72–1.18)	0.5314	**2.35(1.82–3.04)**	**<.0001**	**2.44(1.71–3.48)**	**<.0001**	**0.52(0.37–0.72)**	**0.0001**	1.24(0.95–1.61)	0.1123	**1.39(1.18–1.63)**	**<.0001**	**1.57(1.40–1.76)**	**<.0001**
		Adj	0.98(0.85–1.14)	0.8120	1.00(0.75–1.34)	0.9983	1.02(0.75–1.39)	0.8935	0.97(0.65–1.44)	0.8610	**0.64(0.44–0.92)**	**0.0152**	1.09(0.82–1.46)	0.5535	**1.35(1.12–1.62)**	**0.0015**	**1.24(1.08–1.44)**	**0.0033**
	Upper	Unadj	0.97(0.85–1.11)	0.6451	0.78(0.60–1.01)	0.0634	**1.68(1.28–2.21)**	**0.0002**	**1.80(1.24–2.61)**	**0.0021**	**0.47(0.33–0.67)**	**<.0001**	1.24(0.95–1.61)	0.1109	**2.15(1.85–2.50)**	**<.0001**	**1.31(1.16–1.48)**	**<.0001**
		Adj	1.07(0.92–1.25)	0.3873	0.91(0.67–1.25)	0.5742	1.19(0.86–1.65)	0.2921	0.97(0.64–1.48)	0.8933	**0.59(0.40–0.88)**	**0.0084**	1.11(0.82–1.51)	0.4986	**2.16(1.80–2.59)**	**<.0001**	**1.33(1.14–1.56)**	**0.0003**

COPD = Chronic obstructive pulmonary disease; OR = Odds ratio; 95% CI = 95% confidence interval; ‘-‘ Refer to situation where valid estimate for the Odds Ratio could not be determined owing to insufficient cell values.

**Table 4:** Association (both unadjusted and adjusted) of socio-demographic characteristics with self-perceived specific communicable morbidities, type of ailments and respective care-seeking pattern among recruited residents of Malda, West Bengal, India (N = 43999).

Socio-demographics	Categories	Measurement (Unadj = Bivariate Adj = Multivariate)	Suffering from specific communicable ailments (Based on last 3 episodes of ill-health)								Type of Self-perceived morbidity (most recent)		Care sought from (Ref = Non-qualified)			
			Gastroenteritis		Typhoid		Respiratory tract infection		Skin infections and related disorders		Communicable diseases (Ref = Non-communicable)		Qualified, private sector practitioner		Qualified, Govt. sector practitioner	
			OR (95%CI)	p value	OR (95%CI)	p value	OR (95%CI)	p value	OR (95%CI)	p value	OR (95%CI)	p value	OR (95%CI)	p value	OR (95%CI)	p value
Age group of the subject (Ref = <18–40years)	<5 years	Unadj	**6.14(5.38–7.00)**	**<.0001**	1.03(0.62–1.72)	0.9036	**5.32(4.89–5.79)**	**<.0001**	**2.30(1.87–2.84)**	**<.0001**	**9.83(8.65–11.17)**	**<.0001**	**1.19(1.09–1.31)**	**0.0002**	**0.76(0.65–0.89)**	**0.0005**
		Adj	-	-	-	-	-	-	-	-	-	-	-	-	-	-
	5–<18 years	Unadj	**1.36(1.21–1.54)**	**<.0001**	0.81(0.60–1.09)	0.1647	**1.40(1.31–1.50)**	**<.0001**	**1.37(1.19–1.59)**	**<.0001**	**3.28(3.05–3.54)**	**<.0001**	**0.76(0.71–0.83)**	**<.0001**	0.99(0.89–1.10)	0.8215
		Adj	1.07(0.87–1.32)	0.5394	0.73(0.44–1.20)	0.2141	**1.13(1.01–1.27)**	**0.0357**	0.95(0.74–1.21)	0.6772	**2.51(2.22–2.83)**	**<.0001**	**0.69(0.60–0.78)**	**<.0001**	**0.80(0.68–0.95)**	**0.0112**
	41–60 years	Unadj	**1.52(1.32–1.76)**	**<.0001**	1.07(0.76–1.52)	0.6862	**1.41(1.30–1.54)**	**<.0001**	1.20(1.00–1.45)	0.0559	**0.57(0.53–0.61)**	**<.0001**	**1.36(1.26–1.46)**	**<.0001**	**1.35(1.22–1.49)**	**<.0001**
		Adj	**1.50(1.29–1.75)**	**<.0001**	1.17(0.81–1.70)	0.3968	**1.49(1.36–1.62)**	**<.0001**	**1.27(1.04–1.55)**	**0.0190**	**0.59(0.55–0.64)**	**<.0001**	**1.31(1.21–1.41)**	**<.0001**	**1.29(1.16–1.44)**	**<.0001**
	>60 years	Unadj	**2.46(1.98–3.06)**	**<.0001**	0.43(0.16–1.18)	0.1015	**1.72(1.49–1.99)**	**<.0001**	1.19(0.84–1.70)	0.3286	**0.43(0.38–0.48)**	**<.0001**	**1.78(1.59–1.99)**	**<.0001**	**1.68(1.44–1.96)**	**<.0001**
		Adj	**2.44(1.92–3.11)**	**<.0001**	0.51(0.18–1.43)	0.2016	**1.82(1.56–2.13)**	**<.0001**	1.13(0.77–1.65)	0.5323	**0.44(0.39–0.50)**	**<.0001**	**1.56(1.38–1.78)**	**<.0001**	**1.43(1.20–1.69)**	**<.0001**
Gender (Ref = Male)	Female	Unadj	1.00(0.91–1.09)	0.9256	1.05(0.82–1.34)	0.7115	1.02(0.97–1.08)	0.4132	1.08(0.96–1.22)	0.2088	**0.70(0.66–0.73)**	**<.0001**	0.95(0.90–1.00)	0.0533	**0.85(0.79–0.92)**	**<.0001**
		Adj	1.06(0.93–1.20)	0.3977	1.02(0.76–1.38)	0.8893	1.04(0.97–1.11)	0.2976	1.11(0.95–1.29)	0.1787	**0.72(0.67–0.77)**	**<.0001**	0.95(0.88–1.02)	0.1523	**0.80(0.73–0.88)**	**<.0001**
Religion (Ref = Hindu)	Muslim	Unadj	**1.13(1.03–1.25)**	**0.0105**	**1.63(1.27–2.09)**	**0.0001**	1.04(0.98–1.10)	0.1694	**1.51(1.33–1.71)**	**<.0001**	**1.44(1.36–1.52)**	**<.0001**	**0.83(0.78–0.88)**	**<.0001**	0.97(0.90–1.06)	0.5222
		Adj	**0.86(0.74–0.99)**	**0.0339**	**1.80(1.31–2.46)**	**0.0003**	1.01(0.93–1.10)	0.8205	**1.25(1.06–1.49)**	**0.0089**	**1.18(1.09–1.28)**	**<.0001**	**0.85(0.69–0.76)**	**0.0008**	**0.92(0.87–0.96)**	**<.0001**
	Others	Unadj	0.93(0.41–2.12)	0.8552	-	-	1.41(0.95–2.11)	0.0917	1.59(0.65–3.93)	0.3110	1.39(0.92–2.12)	0.1203	**0.58(0.35–0.95)**	**0.0304**	1.03(0.57–1.85)	0.9272
		Adj	0.76(0.28–2.08)	0.5918	-	-	1.21(0.73–2.00)	0.4602	1.32(0.48–3.64)	0.5892	0.91(0.56–1.49)	0.7080	0.77(0.44–1.35)	0.3569	1.41(0.75–2.65)	0.2819
Caste (Ref = General)	SC/ST/OBC	Unadj	1.03(0.94–1.13)	0.5205	**1.59(1.22–2.08)**	**0.0006**	1.05(1.00–1.11)	0.0669	**0.83(0.73–0.94)**	**0.0028**	**1.12(1.06–1.17)**	**<.0001**	**0.83(0.79–0.88)**	**<.0001**	**1.11(1.02–1.20)**	**0.0136**
		Adj	0.88(0.77–1.00)	0.0500	**1.93(1.40–2.67)**	**<.0001**	1.06(0.98–1.14)	0.1372	0.87(0.75–1.02)	0.0866	**1.15(1.08–1.24)**	**<.0001**	0.97(0.90–1.04)	0.3718	**0.93(0.91–0.96)**	**<.0001**
Education level of the subject (Ref = Illiterate)	Primary	Unadj	**0.79(0.69–0.90)**	**0.0005**	0.91(0.64–1.30)	0.6172	**0.86(0.80–0.94)**	**0.0004**	1.06(0.89–1.27)	0.5146	**1.67(1.56–1.80)**	**<.0001**	1.04(0.96–1.13)	0.2992	0.90(0.82–1.00)	0.0534
		Adj	1.05(0.89–1.24)	0.5975	1.04(0.68–1.58)	0.8734	1.04(0.94–1.14)	0.4901	1.08(0.87–1.34)	0.4960	1.04(0.95–1.14)	0.4108	**1.10(1.00–1.22)**	**0.0461**	0.95(0.84–1.07)	0.4017
	Secondary	Unadj	**0.55(0.48–0.63)**	**<.0001**	0.95(0.67–1.35)	0.7802	**0.75(0.69–0.81)**	**<.0001**	1.02(0.85–1.21)	0.8723	**1.23(1.14–1.32)**	**<.0001**	**1.64(1.52–1.77)**	**<.0001**	0.91(0.82–1.01)	0.0617
		Adj	0.86(0.71–1.03)	0.0960	1.19(0.77–1.83)	0.4408	1.02(0.92–1.13)	0.6868	1.20(0.96–1.51)	0.1082	0.97(0.88–1.07)	0.5397	**1.30(1.18–1.44)**	**<.0001**	0.91(0.80–1.04)	0.1501
	Higher-secondary	Unadj	**0.38(0.27–0.52)**	**<.0001**	0.77(0.40–1.47)	0.4233	**0.69(0.60–0.81)**	**<.0001**	0.78(0.56–1.10)	0.1535	1.08(0.94–1.24)	0.2725	**2.48(2.14–2.86)**	**<.0001**	1.17(0.95–1.44)	0.1500
		Adj	0.78(0.54–1.13)	0.1876	1.20(0.54–2.68)	0.6505	**1.21(1.01–1.46)**	**0.0440**	0.90(0.60–1.35)	0.6090	1.12(0.94–1.34)	0.2057	**1.42(1.19–1.69)**	**0.0001**	1.02(0.79–1.31)	0.8865
	Graduation and above	Unadj	**0.40(0.29–0.56)**	**<.0001**	0.86(0.45–1.65)	0.6540	**0.65(0.55–0.76)**	**<.0001**	**0.44(0.28–0.69)**	**0.0004**	1.00(0.86–1.16)	0.9940	**3.39(2.91–3.95)**	**<.0001**	**0.73(0.55–0.97)**	**0.0302**
		Adj	0.92(0.61–1.41)	0.7084	1.46(0.59–3.59)	0.4109	**1.37(1.10–1.71)**	**0.0054**	0.69(0.40–1.21)	0.1973	**1.38(1.13–1.69)**	**0.0020**	**1.30(1.06–1.59)**	**0.0104**	**0.60(0.43–0.85)**	**0.0036**
Maximum educational level among adult household members (Ref = Illiterate)	Primary	Unadj	**0.81(0.71–0.93)**	**0.0028**	1.10(0.72–1.69)	0.6518	**0.90(0.83–0.98)**	**0.0153**	0.95(0.78–1.15)	0.6103	0.95(0.87–1.03)	0.2020	**1.27(1.15–1.41)**	**<.0001**	0.99(0.87–1.11)	0.8128
		Adj	**0.78(0.66–0.93)**	**0.0058**	1.18(0.72–1.92)	0.5109	**0.85(0.76–0.95)**	**0.0028**	1.02(0.81–1.28)	0.8682	0.92(0.83–1.02)	0.1274	1.07(0.95–1.21)	0.2432	0.97(0.85–1.12)	0.7155
	Secondary	Unadj	**0.64(0.57–0.72)**	**<.0001**	1.22(0.83–1.78)	0.3092	**0.83(0.77–0.90)**	**<.0001**	**0.83(0.70–0.99)**	**0.0368**	**0.80(0.74–0.86)**	**<.0001**	**1.94(1.78–2.12)**	**<.0001**	1.04(0.93–1.16)	0.4663
		Adj	**0.73(0.62–0.87)**	**0.0003**	1.40(0.87–2.24)	0.1621	**0.83(0.75–0.93)**	**0.0006**	0.95(0.76–1.20)	0.6828	0.95(0.86–1.05)	0.3480	**1.26(1.13–1.41)**	**<.0001**	1.03(0.90–1.18)	0.6971
	Higher-secondary	Unadj	**0.42(0.35–0.51)**	**<.0001**	0.82(0.49–1.39)	0.4670	**0.65(0.59–0.73)**	**<.0001**	0.86(0.68–1.08)	0.1891	**0.57(0.51–0.63)**	**<.0001**	**2.80(2.50–3.13)**	**<.0001**	**1.21(1.04–1.40)**	**0.0138**
		Adj	**0.56(0.43–0.72)**	**<.0001**	1.03(0.53–1.97)	0.9378	**0.71(0.62–0.83)**	**<.0001**	1.16(0.87–1.55)	0.3232	0.84(0.73–0.96)	0.0097	**1.40(1.22–1.62)**	**<.0001**	1.05(0.87–1.27)	0.5874
	Graduation and above	Unadj	**0.40(0.33–0.48)**	**<.0001**	0.89(0.54–1.45)	0.6352	**0.54(0.49–0.60)**	**<.0001**	**0.50(0.38–0.64)**	**<.0001**	**0.44(0.40–0.48)**	**<.0001**	**4.56(4.10–5.07)**	**<.0001**	1.09(0.93–1.28)	0.2749
		Adj	**0.56(0.42–0.73)**	**<.0001**	1.15(0.58–2.28)	0.6840	**0.62(0.53–0.73)**	**<.0001**	0.80(0.57–1.12)	0.1948	**0.71(0.62–0.83)**	**<.0001**	**1.91(1.65–2.22)**	**<.0001**	1.00(0.81–1.23)	0.9857
Occupational type (Ref = Sedentary)	Moderate worker	Unadj	0.91(0.80–1.03)	0.1237	1.15(0.84–1.57)	0.3931	**0.83(0.77–0.89)**	**<.0001**	**0.76(0.64–0.88)**	**0.0005**	**0.35(0.33–0.38)**	**<.0001**	**1.12(1.04–1.20)**	**0.0015**	0.97(0.88–1.07)	0.5283
		Adj	1.04(0.86–1.27)	0.6855	1.03(0.64–1.66)	0.9090	0.99(0.89–1.11)	0.8661	**0.73(0.58–0.93)**	**0.0114**	**0.84(0.76–0.94)**	**0.0016**	**0.89(0.80–0.99)**	**0.0304**	**0.79(0.68–0.92)**	**0.0020**
	Hard Worker	Unadj	1.05(0.92–1.18)	0.4784	**1.37(1.01–1.86)**	**0.0447**	**0.93(0.86–1.00)**	**0.0372**	0.87(0.74–1.02)	0.0782	**0.58(0.54–0.62)**	**<.0001**	**0.74(0.68–0.79)**	**<.0001**	0.91(0.82–1.01)	0.0681
		Adj	1.03(0.84–1.26)	0.7789	0.96(0.59–1.54)	0.8578	1.04(0.93–1.16)	0.5490	**0.75(0.59–0.96)**	**0.0197**	0.94(0.85–1.05)	0.3062	**0.72(0.64–0.81)**	**<.0001**	**0.69(0.59–0.81)**	**<.0001**
Residential area (Ref = Urban)	Rural	Unadj	**1.80(1.62–2.00)**	**<.0001**	**2.97(2.14–4.12)**	**<.0001**	**1.22(1.15–1.29)**	**<.0001**	**1.67(1.45–1.91)**	**<.0001**	**1.91(1.81–2.01)**	**<.0001**	**0.53(0.5–0.56)**	**<.0001**	**0.71(0.66–0.77)**	**<.0001**
		Adj	**1.76(1.50–2.07)**	**<.0001**	**2.85(1.86–4.38)**	**<.0001**	**1.27(1.16–1.38)**	**<.0001**	**1.45(1.19–1.77)**	**0.0002**	**1.47(1.36–1.60)**	**<.0001**	**0.82(0.75–0.89)**	**<.0001**	**0.72(0.64–0.81)**	**<.0001**
Water source (Ref = Unsafe)	May be unsafe	Unadj	0.85(0.68–1.07)	0.1697	0.6(0.36–1.02)	0.0578	1.16(1.00–1.35)	0.0511	0.99(0.71–1.37)	0.9488	0.96(0.83–1.11)	0.5619	1.08(0.92–1.27)	0.3678	0.89(0.72–1.10)	0.2944
		Adj	0.88(0.67–1.14)	0.3298	0.89(0.51–1.55)	0.6809	**1.29(1.07–1.54)**	**0.0066**	1.21(0.83–1.77)	0.3139	**1.25(1.05–1.47)**	**0.0101**	0.87(0.72–1.04)	0.1214	0.87(0.69–1.09)	0.2283
	Safe	Unadj	**0.53(0.38–0.73)**	**0.0002**	**0.31(0.12–0.75)**	**0.0097**	**0.77(0.63–0.94)**	**0.0102**	**0.57(0.36–0.91)**	**0.0179**	**0.52(0.43–0.62)**	**<.0001**	**3.47(2.84–4.23)**	**<.0001**	1.32(0.99–1.75)	0.0597
		Adj	0.85(0.56–1.30)	0.4555	0.77(0.26–2.26)	0.6310	1.04(0.81–1.34)	0.7630	1.13(0.66–1.93)	0.6614	1.12(0.89–1.40)	0.3228	**1.33(1.05–1.67)**	**0.0173**	1.04(0.75–1.43)	0.8285
Sanitation level regarding toilet use (Ref = Poor)	Average	Unadj	**0.78(0.70–0.86)**	**<.0001**	0.93(0.70–1.22)	0.5857	**0.91(0.86–0.97)**	**0.0053**	**0.76(0.66–0.87)**	**0.0001**	**0.79(0.74–0.84)**	**<.0001**	**1.67(1.55–1.79)**	**<.0001**	**1.17(1.06–1.28)**	**0.0011**
		Adj	**1.17(1.02–1.34)**	**0.0253**	1.12(0.81–1.54)	0.4905	1.07(0.99–1.16)	0.1089	0.91(0.77–1.07)	0.2523	1.01(0.93–1.09)	0.8903	**1.17(1.07–1.28)**	**0.0004**	1.06(0.95–1.18)	0.3021
	Good	Unadj	**0.51(0.45–0.57)**	**<.0001**	**0.45(0.31–0.65)**	**<.0001**	**0.75(0.70–0.81)**	**<.0001**	**0.57(0.48–0.67)**	**<.0001**	**0.47(0.44–0.51)**	**<.0001**	**3.12(2.90–3.37)**	**<.0001**	**1.22(1.10–1.36)**	**0.0002**
		Adj	0.99(0.82–1.19)	0.8963	0.81(0.50–1.29)	0.3664	0.97(0.87–1.08)	0.5920	0.87(0.69–1.09)	0.2259	**0.78(0.70–0.86)**	**<.0001**	**1.58(1.42–1.75)**	**<.0001**	0.94(0.81–1.08)	0.3918
Socio-economic status (Ref = Very poor)	Poor	Unadj	**0.76(0.66–0.86)**	**<.0001**	0.90(0.62–1.32)	0.5869	**0.86(0.80–0.93)**	**0.0001**	**0.71(0.59–0.85)**	**0.0002**	**0.77(0.71–0.83)**	**<.0001**	**1.76(1.61–1.91)**	**<.0001**	**1.18(1.06–1.32)**	**0.0025**
		Adj	0.94(0.79–1.10)	0.4221	1.01(0.67–1.55)	0.9472	0.97(0.88–1.06)	0.5003	0.82(0.66–1.00)	0.0548	0.98(0.90–1.07)	0.6521	**1.23(1.11–1.36)**	**<.0001**	**1.14(1.01–1.28)**	**0.0421**
	Lower middle	Unadj	**0.65(0.56–0.76)**	**<.0001**	0.98(0.65–1.47)	0.9040	**0.75(0.69–0.82)**	**<.0001**	**0.67(0.54–0.82)**	**<.0001**	**0.73(0.67–0.79)**	**<.0001**	**2.08(1.90–2.28)**	**<.0001**	1.11(0.98–1.25)	0.1190
		Adj	**0.79(0.65–0.95)**	**0.0142**	1.17(0.76–1.81)	0.4706	**0.85(0.77–0.94)**	**0.0024**	**0.78(0.62–0.98)**	**0.0296**	0.95(0.86–1.05)	0.3006	**1.41(1.26–1.57)**	**<.0001**	1.11(0.97–1.28)	0.1424
	Upper middle	Unadj	**0.73(0.64–0.84)**	**<.0001**	1.00(0.69–1.46)	0.9973	**0.67(0.62–0.73)**	**<.0001**	0.68(0.56–0.81)	<.0001	**0.78(0.72–0.85)**	**<.0001**	**2.28(2.09–2.49)**	**<.0001**	0.99(0.87–1.11)	0.8234
		Adj	**0.85(0.72–1.01)**	**0.0657**	0.96(0.64–1.46)	0.8612	**0.73(0.66–0.81)**	**<.0001**	**0.73(0.59–0.91)**	**0.0044**	0.95(0.86–1.05)	0.3002	**1.59(1.43–1.77)**	**<.0001**	1.06(0.92–1.21)	0.4332
	Upper	Unadj	**0.71(0.62–0.81)**	**<.0001**	0.92(0.63–1.36)	0.6827	**0.63(0.58–0.68)**	**<.0001**	**0.79(0.66–0.94)**	**0.0091**	**0.86(0.79–0.93)**	**0.0001**	**1.85(1.69–2.03)**	**<.0001**	0.99(0.88–1.12)	0.9137
		Adj	**0.72(0.60–0.86)**	**0.0004**	**0.63(0.41–0.99)**	**0.0445**	**0.63(0.56–0.70)**	**<.0001**	**0.79(0.64–0.98)**	**0.0304**	**0.84(0.75–0.92)**	**0.0005**	**1.51(1.35–1.69)**	**<.0001**	1.08(0.93–1.25)	0.3061

OR = Odds ratio; 95% CI = 95% confidence interval; ‘-‘ Refer to situation where valid estimate for the Odds Ratio could not be determined owing to insufficient cell values.

Compared to males, females had higher odds of suffering from APD [AOR = 1.60(1.45–1.77)], HTN [AOR = 1.53(1.28–1.83)], anemia [AOR = 16.26(10.75–24.59)] and OA [AOR = 2.58(2.07–3.22)] and lower odds for COPD [AOR = 0.59(0.48–0.73)] and DM [AOR = 0.73(0.57–0.92)]. Muslims suffered less from APD [AOR = 0.77(0.69–0.87)] and gastroenteritis [AOR = 0.86(0.74–0.99)] but more from DM [AOR = 1.40(1.06–1.85)], typhoid [AOR = 1.80(1.31–2.46)] and skin infections [AOR = 1.25(1.06–1.49)] than Hindus. With reference to general, backward castes suffered less from APD [AOR = 0.74(0.67–0.81)], HTN [AOR = 0.82(0.69–0.97)] and anemia [AOR = 0.77(0.60–0.98)] but more from typhoid [AOR = 1.93(1.40–2.67)].

Compared to illiterates, higher familial education was associated with lower likelihood of APD [AOR_Higher Secondary_ = 0.57(0.47–0.70), AOR_≥Graduation_ = 0.57(0.46–0.70)], COPD [AOR_Higher Secondary_ = 0.60(0.41–0.88), AOR_≥Graduation_ = 0.54(0.36–0.81)], anemia [AOR_≥Graduation_ = 0.48(0.26–0.87)], OA [AOR_Higher Secondary_ = 0.61(0.42–0.88), AOR_≥Graduation_ = 0.64(0.44–0.94)], gastroenteritis [AOR_Higher Secondary_ = 0.56(0.43–0.72), AOR_≥Graduation_ = 0.56(0.42–0.73)] and RTI [AOR_Higher Secondary_ = 0.71(0.62–0.83), AOR_≥Graduation_ = 0.62(0.53–0.73)].

Hard workers (reference = Sedentary) were more prone to APD [AOR = 1.45(1.24–1.71)] and anemia [AOR = 1.89(1.17–3.04)] but less vulnerable to COPD [AOR = 0.53(0.40–0.69)] and HTN [AOR = 0.60(0.46–0.77)]. Rural residents, compared to urban, were less likely to have HTN [AOR = 0.54(0.43–0.67)] but more prone to OA [AOR = 1.47(1.15–1.87)], gastroenteritis [AOR = 1.76(1.50–2.07)], typhoid [AOR = 2.85(1.86–4.38)], RTI [AOR = 1.27(1.16–1.38)] and skin infection [AOR = 1.45(1.19–1.77)].

Drinking safer water and practicing better sanitation regarding toilet use seemed to be associated with lower likelihood of suffering from gastroenteritis, typhoid, RTI and skin infections in bivariate analyses but the multivariate analyses lacked power. Relatively higher SES was associated with lower likelihood of anemia [AOR_Upper middle_ = 0.64(0.44–0.92), AOR_Upper_ = 0.59(0.40–0.88)], gastroenteritis [AOR_Upper_ = 0.72(0.60–0.86)], typhoid [AOR_Upper_ = 0.63(0.41–0.99)], RTI [AOR_Upper middle_ = 0.73(0.66–0.81), AOR_Upper_ = 0.63(0.56–0.70)] and skin infections [AOR_Upper middle_ = 0.73(0.59–0.91), AOR_Upper_ = 0.79(0.64–0.98)]. Higher SES also seemed to be associated with higher odds of having HTN [OR_Upper middle_ = 2.35(1.82–3.04), OR_Upper_ = 1.68(1.28–2.21)] and DM [OR_Upper middle_ = 2.44(1.71–3.48), OR_Upper_ = 1.80(1.24–2.61)]. (Tables 3 and 4)

In comparison with respective reference groups, perceived severity of the ailments increased with higher age [for severe disease, AOR_41–60 years_ = 2.34(2.10–2.61), AOR_>60 years_ = 4.25(3.61–5.00)], familial education [for severe disease, AOR_Higher secondary_ = 1.41(1.16–1.72), AOR_>Graduation_ = 1.54(1.26–1.88)], sanitation level regarding toilet use practices [for severe disease, AOR_Good_ = 1.38(1.19–1.61)] and SES [for severe disease, AOR_Upper middle_ = 1.24(1.08–1.44), AOR_Upper_ = 1.33(1.14–1.56)]. Perception of severity was lower among hard-workers [for severe disease, AOR = 0.78(0.67–0.91)] and rural residents [for severe disease, AOR = 0.87(0.77–0.98)]. (Table 3)

With respect to 18–40 year old, younger persons were more likely [AOR_5–18_ = 2.51(2.22–2.83)], and older residents were less likely [AOR_41–60_ = 0.59(0.55–0.64), AOR_>60_ = 0.44(0.39–0.50)] to suffer from communicable diseases (reference = NCD). Compared to respective reference groups, females [AOR = 0.72(0.67–0.77)], residents having higher familial education [AOR = 0.71(0.62–0.83)] and higher SES [AOR = 0.84(0.75–0.92)] had lower likelihood of communicable diseases. Muslims [AOR = 1.18(1.09–1.28)], persons belonging to backward [AOR = 1.15(1.08–1.24)] caste, those who had higher individual education [AOR_≥Graduation_ = 1.38(1.13–1.69)] and rural [AOR = 1.47(1.36–1.60)] residents suffered more from communicable diseases. (Table 4)

With reference to respective comparison groups, subjects aged 5–18 years [AOR_Private_ = 0.69(0.60–0.78), AOR_Govt_ = 0.80(0.68–0.95)], females [AOR_Govt_ = 0.80(0.73–0.88)], Muslim religion [AOR_Private_ = 0.85(0.69–0.76), OR_Govt_ = 0.92(0.87–0.96)], backward caste [AOR_Govt_ = 0.93(0.91–0.96)], physically demanding occupation [for hard work, AOR_Private_ = 0.72(0.64–0.81), AOR_Govt_ = 0.69(0.59–0.81)] and rural residence [AOR_Private_ = 0.82(0.75–0.89), AOR_Govt_ = 0.72(0.64–0.81)] were associated with lower likelihood of visiting qualified practitioners (reference = Non-qualified). Age > 40 years [for 41–60 years age group: AOR_Private_ = 1.31(1.21–1.41), AOR_Govt_ = 1.29(1.16–1.44); for age > 60 years: AOR_Private_ = 1.56(1.38–1.78), AOR_Govt_ = 1.43(1.20–1.69)], higher individual [for higher secondary: AOR_Private_ = 1.42(1.19–1.69) and for ≥ Graduation: AOR_Private_ = 1.30(1.06–1.59)] and familial education [for higher secondary: AOR_Private_ = 1.26(1.13–1.41) and for ≥ Graduation: AOR_Private_ = 1.40(1.22–1.62)], better sanitary practices [for average practice: AOR_Private_ = 1.17(1.07–1.28) and for good practice: AOR_Private_ = 1.58(1.42–1.75)] and higher SES [for Upper middle: AOR_Private_ = 1.59(1.43–1.77) and for Upper: AOR_Private_ = 1.51(1.35–1.69)] were associated with higher odds of seeking care from qualified (reference = Non-qualified) practitioners. (Table 4)

Likelihood of visiting qualified practitioners were lower among subjects who suffered from APD [AOR_Private_ = 0.41(0.37–0.46), AOR_Govt_ = 0.36(0.31–0.43)], OA [AOR_Private_ = 0.72(0.59–0.88), AOR_Govt_ = 0.58(0.43–0.78)], gastroenteritis [AOR_Private_ = 0.28(0.24–0.33), AOR_Govt_ = 0.69(0.58–0.81)], RTI [AOR_Private_ = 0.35(0.32–0.39), AOR_Govt_ = 0.46(0.41–0.52)], skin infections [AOR_Private_ = 0.65(0.55–0.77)]. Those who had COPD [AOR_Private_ = 1.80(1.46–2.23), AOR_Govt_ = 1.78(1.38–2.31)], HTN [AOR_Private_ = 1.94(1.60–2. 36), AOR_Govt_ = 1.37(1.05–1.79)], DM [AOR_Private_ = 4.94(3.55–6.87), AOR_Govt_ = 3.28(2.20–4.91)], typhoid [AOR_Private_ = 2.86(2.04–4.03), AOR_Govt_ = 3.95(2.70–5.79)]and NCDs [AOR_Private_ = 2.31(2.16–2.48), AOR_Govt_ = 1.30(1.18–1.42)] were more likely to visit qualified practitioners. Higher self-perceived disease severity [for moderate: AOR_Private_ = 1.32(1.16–1.51); for severe: AOR_Private_ = 3.16(2.86–3.49), AOR_Govt_ = 1.95(1.71–2.24)] was also positively associated with visiting qualified practitioners. (Table 5)

**Table 5:** Association (both unadjusted and adjusted) of self-perceived specific morbidity type, specific ailments and severity with respective care-seeking pattern among recruited residents of Malda, West Bengal, India (N = 43999).

		Measurement (Unadj = Bivariate Adj = Multivariate)	Care sought from (Ref = Non-qualified)			
			Qualified, private sector practitioner		Qualified, Govt. sector practitioner	
			OR (95%CI)	p value	OR (95%CI)	p value
Type of Self-perceived morbidity (most recent)	Non-communicable diseases (Ref = communicable)	Unadj	**2.31(2.18–2.45)**	**<.0001**	**1.48(1.37–1.60)**	**<.0001**
		Adj	**2.31(2.16–2.48)**	**<.0001**	**1.30(1.18–1.42)**	**<.0001**
Suffering from specific non-communicable ailments (Based on last three episodes of ill-health)	Acid peptic disorder	Unadj	**0.47(0.43–0.52)**	**<.0001**	**0.37(0.32–0.43)**	**<.0001**
		Adj	**0.41(0.37–0.46)**	**<.0001**	**0.36(0.31–0.43)**	**<.0001**
	Chronic obstructive pulmonary disease	Unadj	**1.96(1.62–2.37)**	**<.0001**	**2.10(1.65–2.66)**	**<.0001**
		Adj	**1.80(1.46–2.23)**	**<.0001**	**1.78(1.38–2.31)**	**<.0001**
	Hypertension	Unadj	**3.24(2.72–3.87)**	**<.0001**	**1.82(1.42–2.33)**	**<.0001**
		Adj	**1.94(1.60–2.36)**	**<.0001**	**1.37(1.05–1.79)**	**0.0202**
	Diabetes Mellitus	Unadj	**7.73(5.62–10.64)**	**<.0001**	**4.24(2.87–6.27)**	**<.0001**
		Adj	**4.94(3.55–6.87)**	**<.0001**	**3.28(2.20–4.91)**	**<.0001**
	Anaemia	Unadj	**0.75(0.59–0.94)**	**0.0123**	0.80(0.58–1.09)	0.1603
		Adj	0.84(0.66–1.08)	0.1714	0.94(0.68–1.31)	0.7194
	Osteoarthritis	Unadj	0.84(0.70–1.01)	0.0641	**0.67(0.51–0.88)**	**0.0047**
		Adj	**0.72(0.59–0.88)**	**0.0014**	**0.58(0.43–0.78)**	**0.0003**
Suffering from specific communicable ailments (Based on last 3 episodes of ill-health)	Gastroenteritis	Unadj	**0.33(0.29–0.37)**	**<.0001**	**0.64(0.56–0.74)**	**<.0001**
		Adj	**0.28(0.24–0.33)**	**<.0001**	**0.69(0.58–0.81)**	**<.0001**
	Typhoid	Unadj	**2.53(1.85–3.45)**	**<.0001**	**3.48(2.43–4.97)**	**<.0001**
		Adj	**2.86(2.04–4.03)**	**<.0001**	**3.95(2.70–5.79)**	**<.0001**
	Respiratory tract infection	Unadj	**0.43(0.40–0.46)**	**<.0001**	**0.44(0.40–0.49)**	**<.0001**
		Adj	**0.35(0.32–0.39)**	**<.0001**	**0.46(0.41–0.52)**	**<.0001**
	Skin infections and related disorders	Unadj	**0.63(0.54–0.72)**	**<.0001**	0.84(0.70–1.01)	0.0695
		Adj	**0.65(0.55–0.77)**	**<.0001**	0.84(0.69–1.03)	0.1011
Self-perceived severity (Ref = Mild)	Moderate	Unadj	**1.28(1.15–1.44)**	**<.0001**	1.14(0.97–1.34)	0.1147
		Adj	**1.32(1.16–1.51)**	**<.0001**	1.10(0.92–1.30)	0.2930
	Severe	Unadj	**3.32(3.06–3.61)**	**<.0001**	**2.07(1.84–2.34)**	**<.0001**
		Adj	**3.16(2.86–3.49)**	**<.0001**	**1.95(1.71–2.24)**	**<.0001**

OR = Odds ratio; 95% CI = 95% confidence interval

## Discussion

The socio-demographic distribution of the recruited population in Malda district was typically identical with a developing world poor-resource setting with potential loopholes in healthcare delivery system. The proportion of underprivileged class, poor education, rural residence, sedentary work, poor access to safe water, poor sanitation and overall lower SES rendered the residents of this district vulnerable to morbidity and poor healthcare-seeking.

More than half (55.91%) of the participants suffered from some recent morbidity while respiratory, gastrointestinal and musculoskeletal diseases were most common. This observed burden of self-perceived morbidity was considerably higher than previously reported values (ranged between 27% and 48%) in similar settings.[26–29] Studies conducted in other parts of the globe,[26–28] also indicated that respiratory, gastrointestinal and musculoskeletal ailments were perceived commonly.[26,28,30,31] Probably the chronic and disturbing symptoms of these slowly progressive ailments resulted in more attention. Cardio-vascular diseases were generally reported less as we observed.[26] Burden of reported NCDs was marginally higher than communicable diseases.

More than half of the ailments were treated by non-qualified practitioners, which raised a few concerns. Only about 13% visited qualified physicians from Govt. sector. The scenario seemed similar to that of other parts of India, Vietnam and Bangladesh [26,28,32] but a bit different from Afghanistan and Nepal where majority visited Govt. doctors.[33,34] Easy availability, less fees and better responsiveness were probably in favor of visiting non-qualified practitioners. Alike other settings, among subjects visiting non-qualified practitioners, proportion of communicable diseases were higher compared to NCDs while qualified practitioners from private sector treated more NCDs compared to their counterparts from Govt. sector.[35–37] The results probably indicated towards the lack of provision to quality healthcare services from Governmental sector in these areas, leading to increased inequality in healthcare-seeking. The resultant high burden of out-of-pocket healthcare costs disproportionately affected the poorer population compelling them towards healthcare-seeking from non-qualified practitioners. NCDs probably were given more importance due to their persistent symptoms and the community was probably less confident about the ability of non-qualified practitioners regarding treatment of these diseases.

Among specific ailments, RTI was perceived to be the commonest, followed by APD, gastroenteritis and skin problem. Contrary to some other study, perceived burden of HTN and DM were found to be relatively lower.[29] May be some of the asymptomatic, mild or currently controlled (on medication) cases were missed.

While more than two third subjects considered their ailments as less severe, those who perceived the severity, visited qualified doctors especially in private sector. The perceived severity probably helped them to overcome the potential barriers (may include: cost, transport, availability and waiting time related issues) in better healthcare-seeking.[28,31,34,35,38,39]

Corroborating with prior observation in similar settings elsewhere, children and adolescents were less likely to suffer from NCDs like APD, COPD, HTN, DM, anemia and OA but more from RTI, gastroenteritis and skin infection.[27,33,35,36,40] As evidenced in previous studies, elderly subjects were more prone to APD, COPD, HTN, DM, OA, gastroenteritis and RTI while among adults, risk of these diseases increased with age.[26–29,41,42]

Similar to some previous observation, females had higher likelihood of having APD, anemia and OA but less likely to suffer from COPD and DM [26–28] but gender was not found to be associated with communicable diseases.[33,34,36] Muslims suffered less from APD and gastroenteritis but more from DM, typhoid and skin infections. Subjects belonging to SC/ST/OBC castes suffered less from APD, HTN and anemia but more from typhoid. Probably lower awareness and resultant less attention for milder symptoms did influence the patterns of perceived morbidity.

Supporting some prior evidences [27] and contradicting a few,[26,29] our study indicated that higher household education was probably an important predictor for lowering the risk of APD, COPD, anemia, OA, gastroenteritis and RTI while having more education did not individually help the subjects to suffer less except for COPD. Instead regarding HTN, DM and RTI, corroborating available information, higher individual education was associated with increased morbidity.[43] Compared to individual, household education was probably a stronger predictor for healthy practice and proper decision-making regarding care-seeing, together resulting in less morbidity. On the other hand, for subjects with higher education, sedentary work, occupational pressure and better awareness probably increased the perceived burden of HTN, DM, RTI etc.

Occupation with hard work was associated with higher odds of APD and anemia but lower odds of COPD and HTN. Physical exertion, work environment and appropriate nutrition probably were the key factors. Negative association between physical activity and HTN was well-established in prior studies.[42]

Rural residents compared to urban were less prone to HTN (may be due to environmental factors, less anxiety and stress) but they had higher likelihood of having OA, gastroenteritis, typhoid, RTI and skin infection most likely due to lifestyle related factors, less awareness, poor hygiene and inappropriate sanitation. Urban preponderance of HTN was also reported previously [43] although some researchers did not find significant rural/urban variation.[41]

Drinking safer water was associated with higher perceived burden of HTN and DM. Subjects having better sanitary practices regarding toilet use were also suffering more from APD, HTN, DM and OA. Health awareness and knowledge as probably a confounder here that positively influenced both better practices (regarding drinking safe water, toilet use etc.) and improved perception. Reverse causation might also be a possibility (being diagnosed with the disease resulted in better sanitation and hygiene). Drinking safer water and practicing better sanitation regarding toilet use seemed to be also associated with lower likelihood of suffering from gastroenteritis, typhoid, RTI and skin infections.

Alike prior studies, we also found that, residents having comparatively higher SES were less likely to suffer from anemia, gastroenteritis, typhoid, RTI and skin infections [26,29] but seemed to be having higher odds of having HTN and DM.[27,29] While better SES could have improved awareness and in turn better identification of NCDs, means to prevent communicable diseases were also probably better available to them.

Perceived severity of ailments was higher among those with higher age, better familial education, improved sanitation and upper SES and lower among hard-workers and rural residents had. Higher severity of self-perceived morbidity among elderly was also reported previously.[27] Thus perception of severity also seemed to be driven by awareness and knowledge regarding the ailments.

Compared to those aged between 18–40 years, 5–18 years age group were more likely, and older residents were less likely to suffer from communicable diseases than NCDs. Female gender, better familial education and higher SES were negatively associated with risk of communicable diseases. Muslim religion, backward caste, higher individual education and rural residents had higher odds of suffering from communicable diseases.

Socio-demographic predictors of Healthcare-seeking behavior in our study were quite similar to those reported from other parts of the world as well as India with some variations. While elderly subjects commonly visited qualified private and govt. sector physicians,[34] older children, adolescents and females were less likely to be treated by qualified physicians.[38,39] Although in our study compared to Hindus, Muslims visited qualified practitioners less often, in Nepal, religion was not associated with healthcare-seeking.[36] Backward castes, subjects with physically demanding jobs [26] and rural residents also had lower odds of being treated by qualified practitioners.[35,36,40] Subjects having higher individual and familial education,[26,28,33,36] access to better quality of drinking water, better sanitary practices and higher SES were more likely to visit qualified private practitioners.[26,28,32,34–36,40,44,45] Thus as a whole it was evident that while healthcare-seeking subjects having weaker socio-demographic and economic position had higher likelihood of visiting non-qualified practitioners while extremes of ages were more often treated by qualified ones. Likelihood of visiting qualified doctors in private sector was positively associated with higher socio-economic position and health consciousness.

Subjects suffering from NCDs were more likely to visit qualified practitioners especially the private sector.[37] Alike some prior evidences, patients of APD, OA, gastroenteritis, RTI and skin infections were less likely to be treated by qualified practitioners.[32,44,45] Subjects suffering from COPD, HTN, DM and typhoid had higher likelihood of visiting qualified practitioners. Probably recurrent, short-lasting ailments were not influential enough to pursue the residents to overcome the barriers of better healthcare-seeking while chronic diseases of incurable nature were.

Self-perceived severity of ailments were positively associated with odds of visiting qualified practitioners more so in private sector and this finding also supported prior evidences.[35,36,40] The perception that more severe diseases were worth paying more attention, time and money and thus visiting qualified doctors especially in the private sector probably was reflected here.

Despite efficient sampling design, use of detailed questionnaire and robust analyses, our study had certain limitations. Like any other cross-sectional study, causal interpretation of the observed associations is not recommended. Due to the potential vulnerability to temporal ambiguity by design, some of our observations might have suffered from reverse causation. Although self-perceived morbidity and severity are currently being considered an efficient parameter for the estimation of health needs in communities worldwide, keeping the lower literacy and potential lack of awareness in mind, the reported self-perceived morbidity pattern should only be interpreted as perceived health need of the community, not the prevalence. Residual confounding due to variables not included in our analyses could also be an issue. Information bias due to misclassification of self-reported information should always be kept in mind, especially due to the potential for differential recall. But we do not consider those to be serious issues here because we only dealt with the recent ailments, hence recall period was short and in majority of cases, medical records were consulted. Although results of our study should be extrapolated beyond the study sample with caution, still we are not worried about the generalizability of our results due to the representative nature of our study sample and very low (<8%) non-response.

## Conclusion

In this poor-resource setting, most important predictor for healthcare-seeking was the perception regarding severity and nature of ailments, while age, gender, caste, religion, familial education, SES, residential area, sanitation and hygiene influenced the morbidity pattern and relevant healthcare-seeking. Keeping the high burden of self-perceived morbidity in mind, interventions to improve physical health, awareness and care-seeking practices targeting children, elderly, females, backward castes, minority groups, illiterates, rural residents and those having lower SES, poor sanitary practices and inadequate access to safe drinking water were required urgently. Simultaneously, efforts to improve the healthcare service delivery might consider implementation of intervention targeting improvement of knowledge and practice among non-qualified practitioners in poor-resource settings where seeking healthcare services from these practitioners seemed to be a common occurrence.
